# Photoelectrocatalytic Degradation of Paraquat by Pt Loaded TiO_2_ Nanotubes on Ti Anodes

**DOI:** 10.3390/ma11091715

**Published:** 2018-09-13

**Authors:** Levent Özcan, Turan Mutlu, Sedat Yurdakal

**Affiliations:** 1 Biyomedikal Mühendisliği Bölümü, Mühendislik Fakültesi, Afyon Kocatepe Üniversitesi, Ahmet Necdet Sezer Kampüsü, 03200 Afyonkarahisar, Turkey; 2 Kimya Bölümü, Fen-Edebiyat Fakültesi, Afyon Kocatepe Üniversitesi, Ahmet Necdet Sezer Kampüsü, 03200 Afyonkarahisar, Turkey; turan.mutlu@redokslab.com

**Keywords:** photoelectrocatalysis, TiO_2_ nanotube, Pt loaded TiO_2_, paraquat, polar herbicide, degradation

## Abstract

Nanotube structured TiO_2_ on Ti surface were prepared in ethylene glycol (Ti/TiO_2_NTEG) medium by anodic oxidation method with different times and then the plates were calcinated at different temperatures. Non-nanotube structured Ti/TiO_2_, prepared by thermal oxidation method, and nanotube structured TiO_2_ on Ti plate in hydrogen fluoride solution were also prepared for comparison. Pt loaded Ti/TiO_2_NTEG photoanodes were also prepared by cyclic voltammetry method with different cycles and the optimum loaded Pt amount was determined. Photoanodes were characterized by using X-ray Diffraction (XRD), Scanning Electron Microscopy-Energy-Dispersive X-ray Analysis (SEM-EDX), and photocurrent methods. XRD analyses proved that almost all TiO_2_ is in anatase phase. SEM analyses show that nanotubes and Pt nanoparticles on nanotube surface are dispersed quite homogeneously. The longest nanotubes were obtained in the ethylene glycol medium and the nanotube length increased by increasing applied anodic oxidation time. In addition, a linear correlation between nanotube length and XRD peak intensity was found. Moreover, SEM-EDX and XRD analyses evidence that Pt nanoparticles on nanotube surface are metallic and in cubic structure. Photoelectrocatalytic degradation of paraquat was performed using the prepared photoanodes. Moreover, electrocatalytic and photocatalytic degradations of paraquat were also investigated for comparison, however lower activities were observed. These results evidence that the photoanodes show a significant synergy for photoelectrocatalytic activity.

## 1. Introduction

Water pollution caused by organic pollutants (i.e., herbicides, pesticides, pharmaceuticals and care products) is a major problem which should be faced. Some hydrophobic organic compounds could accumulate in oil or similar mediums in which they are very stable and could cause public health problems [1]. Therefore, their elimination needs great efforts. In recent years, the production and application of herbicides and pesticides have been changed; polar and more easily degraded ones have been replaced by nonpolar and stable ones [2].

**Figure 1:**
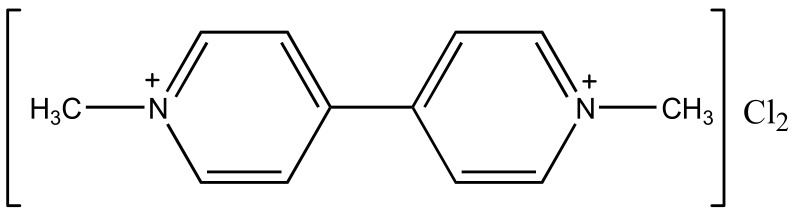
The chemical structure of paraquat (1,1′-dimethyl-4,4′-bipyridinium dichloride).

Paraquat (1,1′-dimethyl-4,4′-bipyridinium dichloride) is a pyridylium herbicide and it is highly used worldwide. Figure 1 shows the chemical structure of paraquat; its polarity and therefore its solubility in water is very high (620 g/L). Although paraquat is prohibited by the European Union, it is still used in almost ninety countries. The high toxicity of paraquat has serious harm to human health and the environment, thus it is important to seek an effective degradation process [3].

The chemical oxidation method using compounds such as chlorine, hydrogen peroxide and ozone rarely provides total mineralization of water pollutants. Even though biological oxidation is economical, the presence of poisonous and heat-resistant pollutants in water makes this method insufficient. Resistant organic compounds could be removed by conventional methods such as ultrafiltration and adsorption techniques. However, the main drawback of these methods is that the pollutants pass from one phase to another one without degradation [4].

Photoelectrocatalytic (PEC) techniques could be an effective environmentally friendly alternative for pollutant degradation as it could be performed by using water as solvent, oxygen from air as oxidant and without additional toxic chemicals [5]. TiO_2_ is the most used photocatalyst because of its high photocatalytic (PC) activity, biological and chemical stability, low cost, non-toxic formation and non-photocorrosive nature [6,7,8]. Although the PC process, in which a semiconductor is used as the catalyst and ultraviolet (UV) or ultraviolet-visible (UV-Vis) irradiation as an energy source is a common method for elimination of harmful compounds, their application is limited due to their low quantum yield caused mainly by high recombination rate [4]. Photoelectrocatalysis, i.e., the combination of heterogeneous photocatalysis with electrocatalysis, is an effective tool for hindering the photogenerated pairs recombination and consequently for improving the yield of reactions [8,9]. This method involves the application of an external potential bias to a thin TiO_2_ layer deposited on a conductive support [8,10,11,12]. In other words, under suitable irradiation (i.e., UV or UV-Vis), the used bias allows the electrons to migrate across the electrode and the separation of electron (e^−^) and hole (h^+^) pairs is improved, thus increasing the probability of reaction occurring at the working electrode surface.

Nowadays, nanostructured TiO_2_ is frequently used in PEC studies in many different areas such as degradation of harmful compounds [13,14], water splitting reactions [15,16] or partial oxidations [9,17,18] due to its high surface area and good electron transport characteristics [19].

The first synthesis of TiO_2_ by anodic oxidation was investigated with basic peroxide and chromic acid treatment by Assefpour-Dezfuly and coworkers [20]. Zwilling and coworkers [21] investigated nanoporous titanium dioxide in fluoride-containing electrolyte, pioneering a big advancement in this area over the last two decades. Many researchers working in this area have made great efforts to find the optimum electrolyte conditions and experiment parameters to obtain high quality and self-assembled titanium dioxide nanotube array. Gong et al. [22] prepared self-assembled TiO_2_ nanotubes with the anodization of Ti plate in H_2_O/HF electrolyte medium at room temperature, even though the nanotube length was limited to a few hundred nanometers. Several micrometer length TiO_2_ nanotubes have been prepared in neutral electrolytes containing fluoride ions such as Na_2_SO_4_/NaF or (NH_4_)_2_SO_4_/NH_4_F [23,24]. During the anodization process, undulatory and ring shapes were observed in the nanotube TiO_2_ walls obtained by current fluctuation. In subsequent works, anodization was carried out in organic electrolytes such as formamide, dimethylsulfoxide, ethylene glycol (EG) or diethylene glycol containing fluoride to produce smooth and several-hundred-micrometer length nanotube TiO_2_ [25,26].

The loading of noble metal nanoparticles such as Au [27], Ag [28], Pd [29] and Pt [30] on the TiO_2_ nanotube surface is an effective tool to increase its (photo)catalytic properties. It is evidenced that modification of photocatalysts with the noble metals improves the PC and PEC activities by preventing the recombination rate of the electron–hole pairs [31].

In a previous study, we investigated the PEC synthesis of *p*-anisaldehyde from 4-metoxybenzyl alcohol in water, under UV irradiation [9] by using Ti/TiO_2_ photoanodes prepared both by thermal oxidation and dip-coating methods. The photoanodes prepared by thermal oxidation showed better aldehyde selectivity and activity performance than dip-coated ones.

For the first time, we reported the selective PEC oxidation of 5-(hydroxymethyl)-2-furaldehyde to 2,5-furandicarbaldehyde by nanotube structured TiO_2_ on Ti layer as photoanodes, prepared by anodic oxidation method in HF medium [18]. In addition, the photoanodes were also platinized by photoreduction method and both product selectivity and PEC activity significantly increased.

Althought some PC paraquat degradation works have been reported [1,32,33], in the present work, PEC degradation of paraquat was investigated for the first time. Highly active nanotube structured TiO_2_ on Ti layers as photoanodes was prepared by anodic oxidation method in EG medium and used in paraquat degradation. The effects of anodic oxidation time on nanotube length and the amount of Pt loading by cyclic voltammetry were also investigated. Photoanodes prepared by thermal oxidation (Ti/TiO_2_-500) [9] and anodic oxidation in HF medium (Ti/TiO_2_-NTHF-X) [18] were used for comparison.

## 2. Materials and Methods

### 2.1. Preparation of Ti Plates

Ti plates (5.0 cm × 8.0 cm × 0.10 cm) were used for the photoanode preparation. The plates were grinded (800, 1000, 1200 and 1500 grids of emery papers, respectively) to smooth the surface, and then sonicated in acetone, ethanol and water (10 min for each solvent). The Ti plates were chemically cleaned in a solution medium containing 4% HF, 31% HNO_3_ and 65% H_2_O for 30 s. Then, the Ti plates were again sonicated in water for 10 min and dried at room temperature.

### 2.2. Preparation of Thermally Oxidized Photoanode (Ti/TiO_2_)

The photoanode was produced by oxidizing the Ti plate surfaces by means of thermal annealing in air (temperature increasing rate: 3 °C/min) at 500 °C for 3 h in an oven (PLF-110/10 model, Protherm Furnaces, Ankara, Turkey). The obtained sample was labeled as Ti/TiO_2_-500, where 500 indicates the thermal treatment temperature expressed in °C. Figure S1 shows the photo of Ti/TiO_2_-500 photoanode.

### 2.3. Preparation of Nanotube Structured TiO_2_ Photoanodes by Anodic Oxidation in Hydrogen Fluoride Solution (Ti/TiO_2_NTHF-X)

Ti plate immersed in an aqueous solution containing 0.15 M HF [18] were subjected to an anodic oxidation process by applying for different times a 20 V potential with a direct current (DC) power supply device (Meili, MCH-305D-II model, MCH Instrument, Shenzhen, China). Thus, nanotube structured amorphous TiO_2_ was formed on the Ti plate surface. Figure S2 shows experimental setup system used for the anodic oxidation. In this system, carbon felt electrode was used as the cathode. The carbon felt was immersed in a 1 M HNO_3_ aqueous solution for one day to make its surface hydrophilic. In addition, the process was carried out at 200 rpm and at 18 °C (±2 °C), in a high-density polyethylene container, instead of glass, because of the use of fluoride containing solutions.

Figure S3 shows the electrodes prepared by anodic oxidation and thermally treated at different temperatures (400–700 °C, temperature increasing rate: 3 °C/min). These photoanodes were named as Ti/TiO_2_NTHF-X-Y, where “X” refers to the anodic oxidation time (in hours) and “Y” the calcination temperature.

### 2.4. Preparation of Nanotube Structured TiO_2_ Photoanodes by Anodic Oxidation in Ethylene Glycol Solution (Ti/TiO_2_NTEG-X)

The solution used for the anodic oxidation was prepared by dissolving NH_4_F in a concentration of 0.3% (*w*/*w*) in a solution containing 2% (*v*/*v*) water and 98% (*v*/*v*) EG [34]. Initially, a 60 V potential was used [34], as the nanotubes exfoliated the voltage was decreased to 40 V. The formed TiO_2_ nanotubes were in amorphous phase. To transform them to crystalline one, the electrodes were calcined at 450, 500 or 550 °C (temperature increasing rate: 3 °C/min). The prepared electrodes were named as Ti/TiO_2_-NTEG-X-Y, where “X” refers to the applied voltage time (in hours) for anodic oxidation in EG and “Y” the heat treatment temperature (°C). The photographs of the photoanodes prepared by this method are shown in Figure S4.

### 2.5. Pt Loading on Ti/TiO_2_NTEG-3h-500 Photoanodes

Pt loading on Ti/TiO_2_NTEG-3h-500 electrode was carried out by using a cyclic voltammetry (CV) technique with an aqueous solution of 0.25 mM H_2_PtCl_6_·6H_2_O and 25 mM H_2_SO_4_. The Pt^4+^ ions in solution were electrochemically loaded on the TiO_2_ nanotube surface with different cycle numbers (voltage range: −0.4–+0.5 V, scan rate: 10 mV/s). Ag/AgCl (3M KCl) electrode was used as the reference electrode and a Ti plate (5.0 cm × 8.0 cm × 0.10 cm) was used as the counter electrode. Figure S5 shows voltammograms obtained during Pt nanoparticle loading on Ti/TiO_2_NTEG-3h-500 electrode by CV until 4 cycles. The peak at ca. −0.35 V decreased by increasing the cycle number probably due to the decrease of Pt^4+^ ions concentration in solution.

### 2.6. Photoanode Characterization

X-ray diffraction (XRD) patterns were recorded by a Shimadzu (XRD-6000 model) diffractometer (Shimadzu Corporation, Tokyo, Japan) by using a Cu Kα radiation (1.544 A°) and a 2Ɵ scan rate of 1.281 degree/min^−1^.

Scanning electron microscope (SEM) images were obtained by using a FEI microscope (NanoSEM 650 model, FEI Company, Hillsboro, OR, USA). SEM images were obtained by using TLD detector. EDX spectra were taken with the EDX detector available in the SEM system. In addition SEM images of Figures 4–6 and Figure S6 were obtained by another SEM instrument (Carl Zeiss ULTRA Plus, Germany).

The performances of the photoanodes were determined chronoamperometrically under UV irradiation in the same electrolyte medium used for PEC paraquat degradation.

### 2.7. Photo-, Electro- and Photoelectro-Reactivity Set up and Procedure

PEC and EC experiments of paraquat degradation were carried out with a three-electrode electrochemical system connected to a computer-controlled potentiostat-galvanostat (Ivium, CompactStat model, Ivium Technologies, Eindhoven, The Netherlands) device. In this system, Ti/TiO_2_ photoanodes, Pt electrode (gauze) and Ag/AgCl (3 M KCl) electrodes were used as working, counter and reference electrodes, respectively. The sizes of the photoanode immersed part were 5.0 cm × 6.0 cm × 0.10 cm. In the PEC and PC experiments, 6 UV fluorescent lamps (8 W) with a maximum wavelength of 365 nm were used as the irradiation source. The PEC and EC experiments setup system is shown in Figure S7. The distance between the photoanodes and the light source is 7 cm, and the light intensity at this distance from the photoanode surface is about 13 W/m^2^. This value was determined by a radiometer (DO9721, Deltaohm, Caselle di Selvazzano, Italy) with a probe measuring 315–400 nm.

Experiments were carried out in environmentally friendly conditions using water as the solvent and oxygen in the air as the oxidant. The solution was in contact with atmospheric air, therefore the concentration of dissolved oxygen was always assumed to be in equilibrium with oxygen of the atmosphere.

The initial concentration of paraquat was 37.4 μM and the concentration of Na_2_SO_4_ used as electrolyte was 50 mM. A Pyrex beaker of 150 mL was used as the reactor for degradation of 150 mL of paraquat solution.

Before switching the lamp on, the solution was stirred for 10 min at room temperature under dark condition without using any voltage to reach the thermodynamic equilibrium. During the runs, the solution was maintained at ca. 25 °C.

### 2.8. Analytical Techniques

Samples were withdrawn at fixed time intervals (0, 30, 60, 120 and 180 min) and analyzed by UV-Vis spectrophotometer (UV-1700 model, Shimadzu Corporation, Tokyo, Japan). A linear calibration graph was obtained from the absorbance values of standard solutions of paraquat. The conversion values (%) were calculated as follows:

% Conversion = [(concentration of reacted paraquat)/(initial concentration of paraquat)] × 100

All used chemicals were purchased from Sigma Aldrich (Darmstadt, Germany) with a purity >98.0%.

## 3. Results and Discussion

### 3.1. Characterization Results

**Figure 2:**
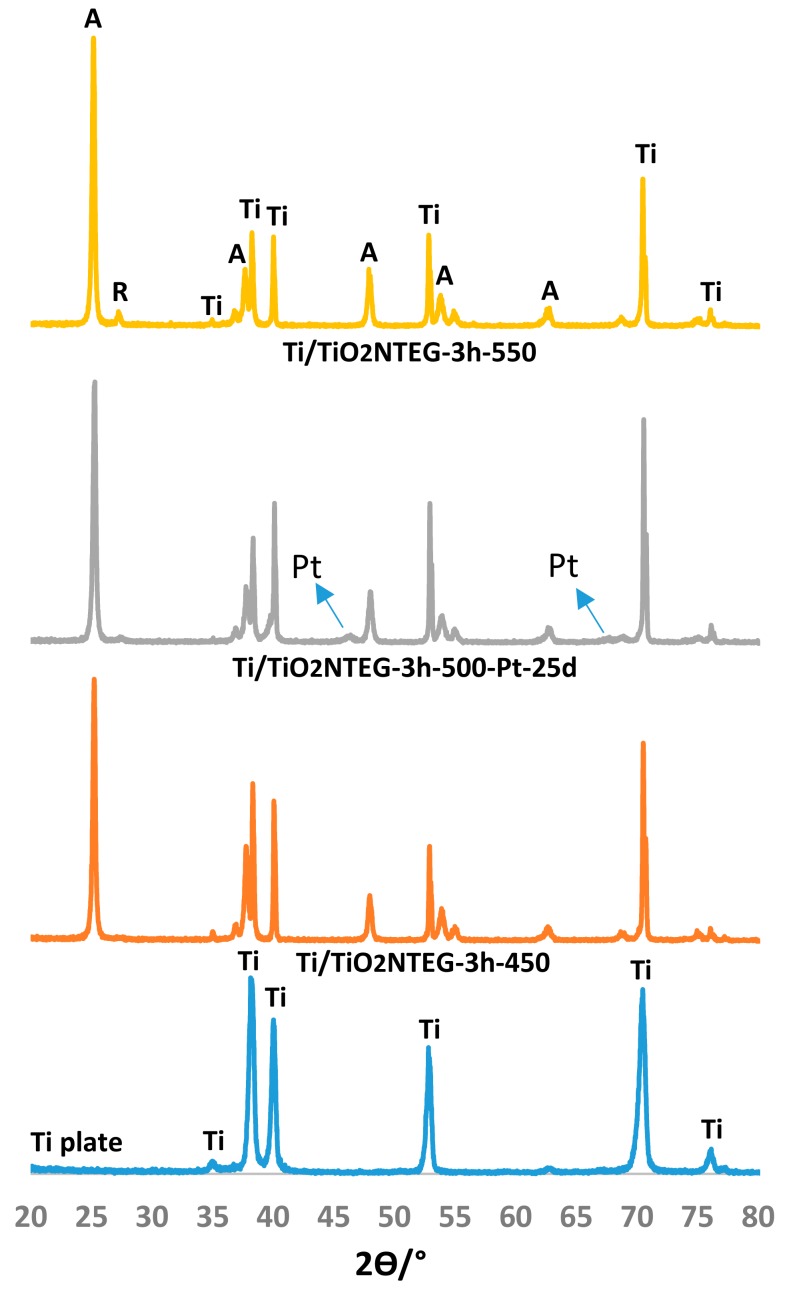
XRD patterns of the Ti/TiO_2_-NTEG-3h-Y photoanodes.

**Figure 3:**
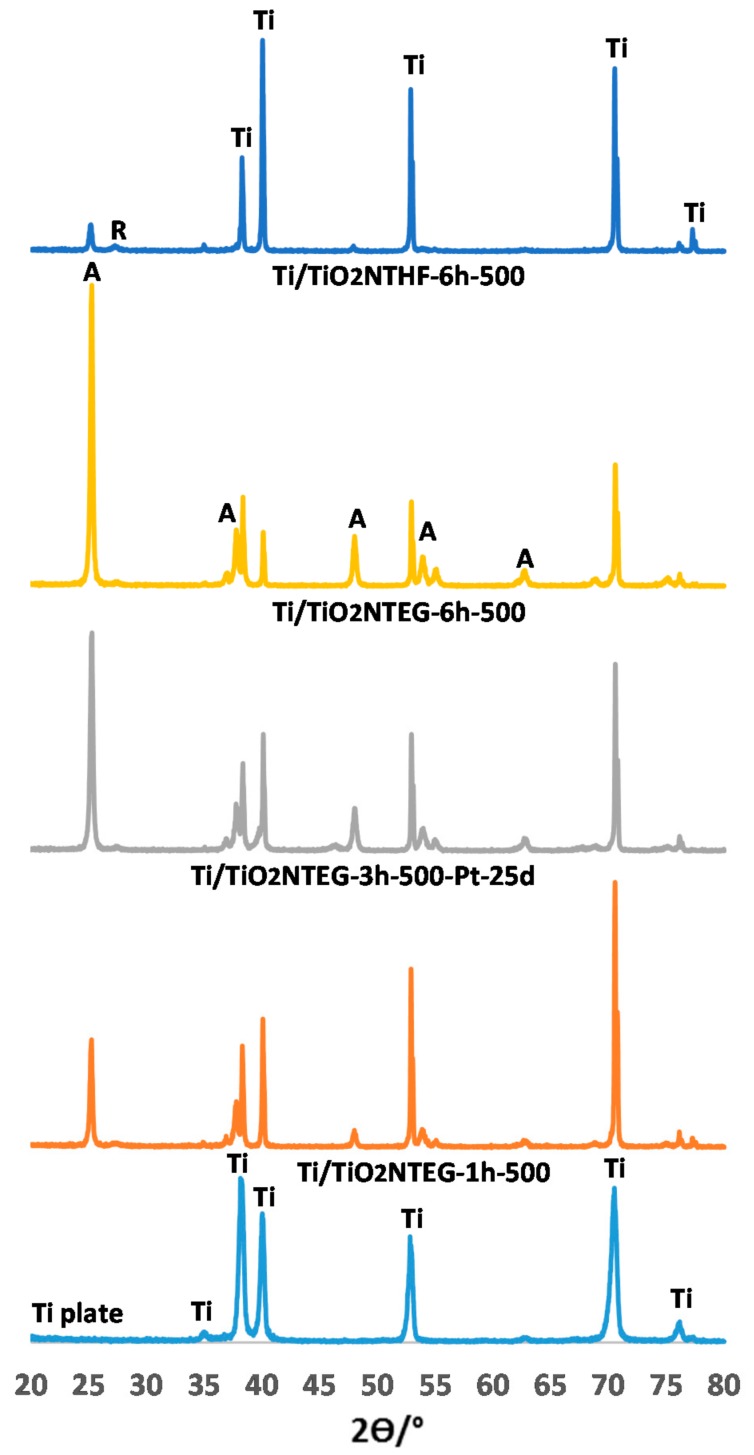
XRD patterns of the Ti/TiO_2_-NTEG-Xh-500 photoanodes.

Figure 2 and Figure 3 show XRD patterns of nanotube TiO_2_ supported photoanodes. XRD patterns of pristine Ti layer are also given as comparison. The figures show peaks at 2Ɵ = 25.58°, 38.08°, 48.08° and 54.58° that belong to anatase phase; peaks at 2Ɵ = 27.5°, 36.5°, 41.0°, 54.1° and 56.5° that belong to rutile phase [35]; and peaks at 2Ɵ = 34.95°, 38.25°, 40.05° and 52.90° that belong to metallic Ti [18]. In addition, the peak values at 2Ɵ = 39.8 °, 46.2 ° and 67.5 ° refer to metallic platinum [18].

Figure 2 shows XRD patterns of Ti/TiO_2_NTEG-3h-450, Ti/TiO_2_NTEG-3h-500-Pt-25cycles and Ti/TiO_2_NTEG-3h-550 prepared in the EG medium and calcined at different temperatures (450–550 °C). The prepared photoanodes contain bands of TiO_2_ mainly in anatase phase and of metallic Ti, whereas only traces of rutile were found. It can be noticed that the peak intensity of Ti decreases by increasing the calcination temperature, while the peak intensity of anatase increase. The most thermodynamically stable phase of TiO_2_ is rutile [36], therefore its peak intensity increases by increasing the heat treatment temperature.

Pt was not observed for the sample Ti/TiO_2_NTEG-3h-500-Pt-4cycles since Pt amount is not enough to be determined by XRD analysis. On the contrary, XRD analysis of Ti/TiO_2_NTEG-3h-500-Pt-25cycles sample show that the loaded Pt is in metallic form (Figure 2 and Figure S8). 

Figure 3 shows the XRD patterns of nanotube structure of TiO_2_ on Ti prepared in the EG medium and for different anodic oxidation times (1, 3 and 6 h), and then calcined at 500 °C. XRD of the Ti/TiO_2_-NTHF-6h-500 electrode, prepared in the HF solution, was added for comparison purposes. TiO_2_ in all photoanodes is mainly in anatase with trace amount of rutile phase. The XRD peak intensities of anatase phase significantly increased by increasing anodic oxidation time used for photoanode preparation. Since all these photoanodes were calcined at the same temperature (500 °C), the crystallization degree of anatase phase of the electrodes is similar. Moreover, the increase in peak intensity of anatase phase (peak area values in Table 1) could be related to the amount of TiO_2_ on the Ti surface, and thus to the length of the nanotubes. On the contrary, by increase the nanotube lengths, the XRD peaks of metallic Ti decrease. The XRD peak intensity of the electrode prepared by anodic oxidation method in the HF solution (Ti/TiO_2_NTHF-6h-500) is very low compared to the others prepared in EG, indicating that the length of the TiO_2_ nanotubes of Ti/TiO_2_NTHF-6h-500 is also shorter.

Table 1 reports the crystal phases of the photoanodes, the peak areas (101) of anatase and rutile phases, and the primary particle sizes determined from the Scherrer equation. The primary particle sizes of the anatase phase of all the electrodes are close to each other (ca. 35 nm). This value is approximately the same as the wall thickness of TiO_2_ nanotubes (see Table 2). Since the rutile peak areas are very low, their results are not reliable enough (range from 23 to 43 nm). The primary particle size of metallic Pt of Ti/TiO_2_NTEG-3h-500-Pt-25cycles electrode is 28 nm.

According to SEM image (Figure S9) of Ti/TiO_2_-500, TiO_2_ on the surface of the Ti plate is in the form of a slightly rough film [18].

SEM image of Ti/TiO_2_NTHF-1h-500 is shown in Figure S10. The nanotubes prepared by this method showed an independent formation with each other [18]. In other words, there are spaces between the nanotubes and each nanotube does not have a common wall. The values of tube length, wall thickness and tube diameter of nanotubes are reported in Table 2. The nanotubes are distributed homogeneously on the surface, the thickness of the nanotube wall is ca. 14 nm and the diameter of the tube hole is ca. 90 nm.

**Figure 4:**
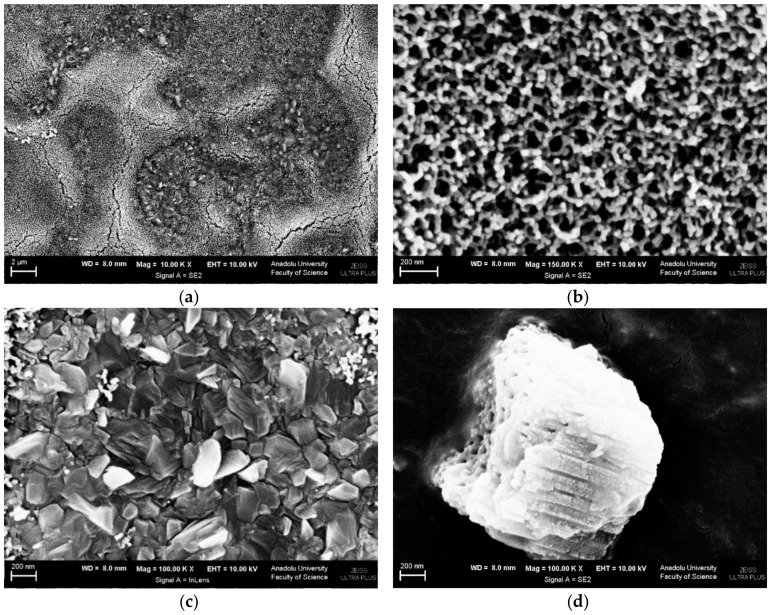
SEM images of Ti/TiO_2_NTHF-6h-650 photoanode: (**a**) top view (magnification: 10,000×), (**b**) top view (magnification: 150,000×), (**c**) top view (magnification: 100,000×) and (**d**) cross-section view (magnification: 100,000×).

SEM images of Ti/TiO_2_NTHF-6h-650 are shown in Figure 4 (and Figure S6). It can be noticed that the nanotubes cover only some part of the surface. This aspect is probably due to the higher calcination temperature. Indeed, in Figure 4a, it is possible to distinguish two zones, a clearer one in high ground which refers to TiO_2_ in nanotubes (Figure 4b) and a darker one that can be attributed to a well crystallized TiO_2_ in layer (Figure 4c) with particle sizes of ca. 200 nm. In addition, its wall thickness is higher and internal diameter lesser than Ti/TiO_2_NTHF-6h-500. Total thickness of both values of both plates are almost the same (ca. 105 nm).

A cross-sectional SEM image of the Ti/TiO_2_NTHF-6h-650 (Figure 4d) allowed determining the nanotube length (ca. 1 μm).

**Figure 5:**
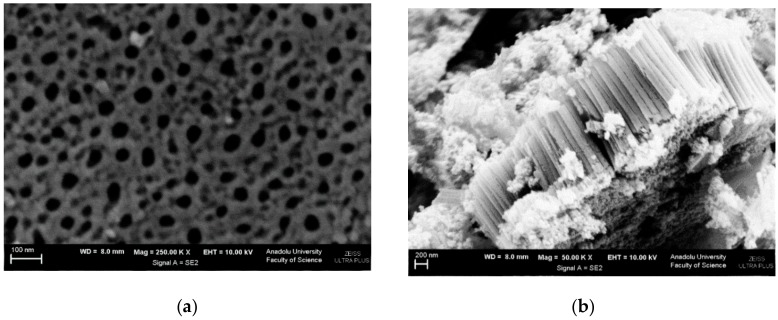
SEM images of Ti/TiO_2_NTEG-1h-500 photoanode: (**a**) top view (magnification: 250,000×) and (**b**) cross-section view (magnification: 50,000×).

Figure 5 shows SEM images of Ti/TiO_2_NTEG-1h-500 electrode. Figure 5a indicates a good distribution of the nanotubes on the surface, whereas Figure 5b shows the cross-section image of the nanotubes. It was possible to determine the wall thickness of the nanotubes (35 nm), the diameter of the tube hole (43 nm), and the average nanotube length (1.7 μm).

**Figure 6:**
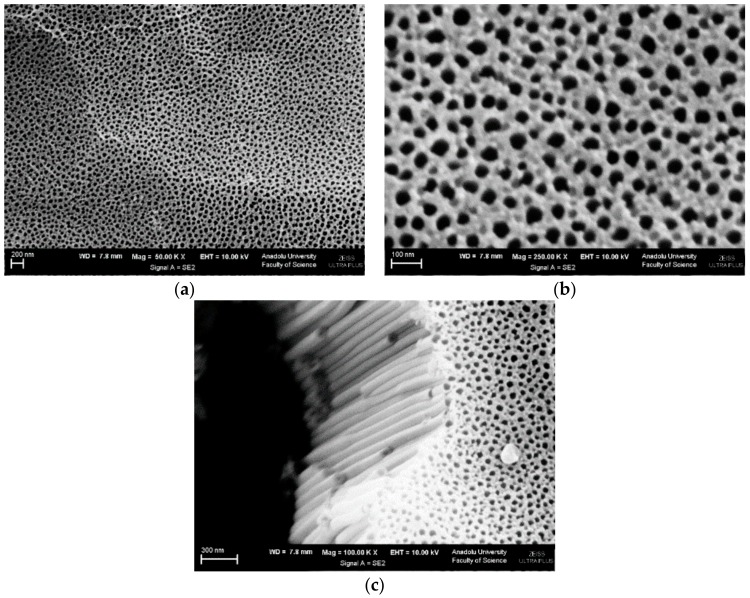
SEM image of Ti/TiO_2_NTEG-2h-500 photoanode: (**a**) top view (magnification: 50,000×), (**b**) top view (magnification: 250,000×) and (**c**) cross-section view (magnification: 100,000×).

SEM images of the Ti/TiO_2_NTEG-2h-500 photoanode are shown in Figure 6. Figure 6a,b show the same image with different magnifications, while Figure 5c shows the cross-section of the material. The nanotubes are distributed quite homogeneously on the surface of the Ti sheet. It can be noticed (Figure 6c) that the nanotube formation occurs even at the bottom of the tubes. However, the inner diameter of the nanotubes in the bottom part is narrower than the top. This result shows that nanotubes form from outside to inside.

**Figure 7:**
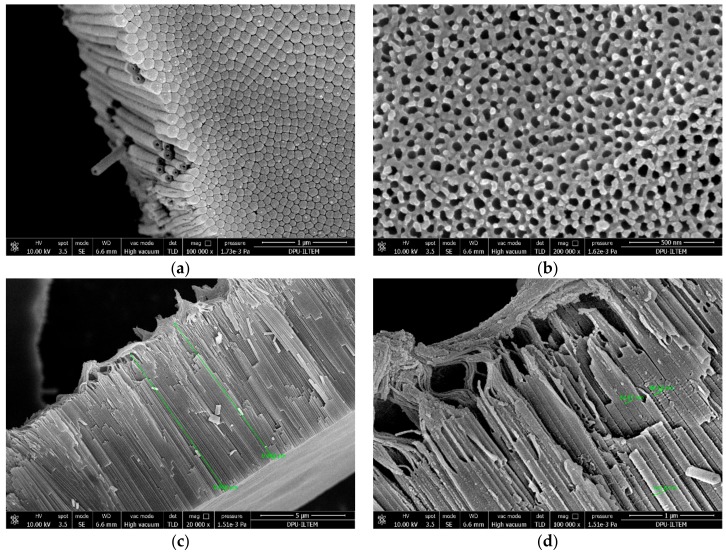
SEM images of Ti/TiO_2_NTEG-4h-500 photoanode: (**a**) bottom view (magnification: 100,000×); (**b**) top view (magnification: 200,000×); (**c**) cross–section view (magnification: 20,000×); and (**d**) cross–section view (magnification: 100,000×).

Figure 7 (and Figure S11) shows SEM images of the bottom, top and cross-section of Ti/TiO_2_NTEG-4h-500 photoanode. Ti surface is completely covered with nanotubes, as shown in Figure 7a. Figure 7a,b shows a quite homogeneous distribution of the nanotubes. Figure 7c,d and Figure S11 are different magnified cross–sections of Ti/TiO_2_NTEG-4h-500 photoanode. The length of the nanotube is about 9.8 μm, and this size extends over a wide range (Figure S11). This nanotube length is greater than the Ti/TiO_2_NTEG-1h-500 photoanode. Some nanotubes zones are covered by TiO_2_ sheets (see Figure 7d). The nanotube wall thickness and hole diameter of tube values are close to the Ti/TiO_2_NTEG-1h-500 electrode (ca. 35 and ca. 47 nm, respectively). However, the wall thickness is thicker at the bottom of the tube, while the hole diameter appears to be smaller (~38 and ~27 nm, respectively, Table 2).

**Figure 8:**
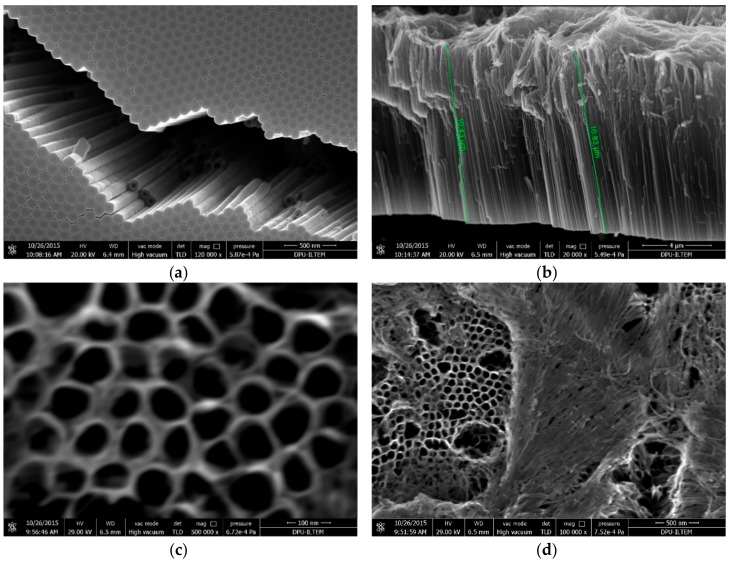
SEM images of Ti/TiO_2_NTEG-6h-500 photoanode: (**a**) bottom view (magnification: 120,000×); (**b**) cross–section view (magnification: 20,000×); (**c**) top view (magnification: 500,000×); and (**d**) top view (magnification: 100,000×).

Figure 8 shows some SEM images of Ti/TiO_2_NTEG-6h-500 photoanode from bottom (Figure 8a and Figure S12b), cross–section (Figure 7b and Figure S12a) and top view (Figure 8c,d). From observation of Figure 8b, it is possible to determine that the nanotube length of this electrode is the longest one (ca. 11 μm). The data in Table 2 show that there is a linear relationship between the anodic oxidation time for the preparation and the nanotube length. Similar relationship was also observed from the peak intensities in XRD analyses. Generally, nanotubes are uniformly distributed on the layer surface (Figure 8c), but there are also some regions covered by TiO_2_ sheets (Figure 8d). This behavior was found for long anodic oxidation times. The wall thickness of the nanotubes for this sample is thinner than the others (ca. 20 nm) and the inner hole diameter is larger (~80 nm, Table 2).

**Figure 9:**
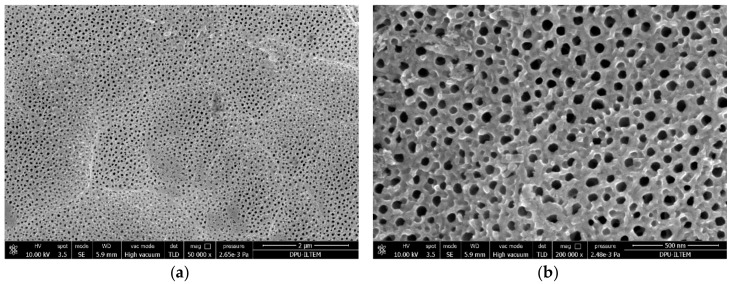

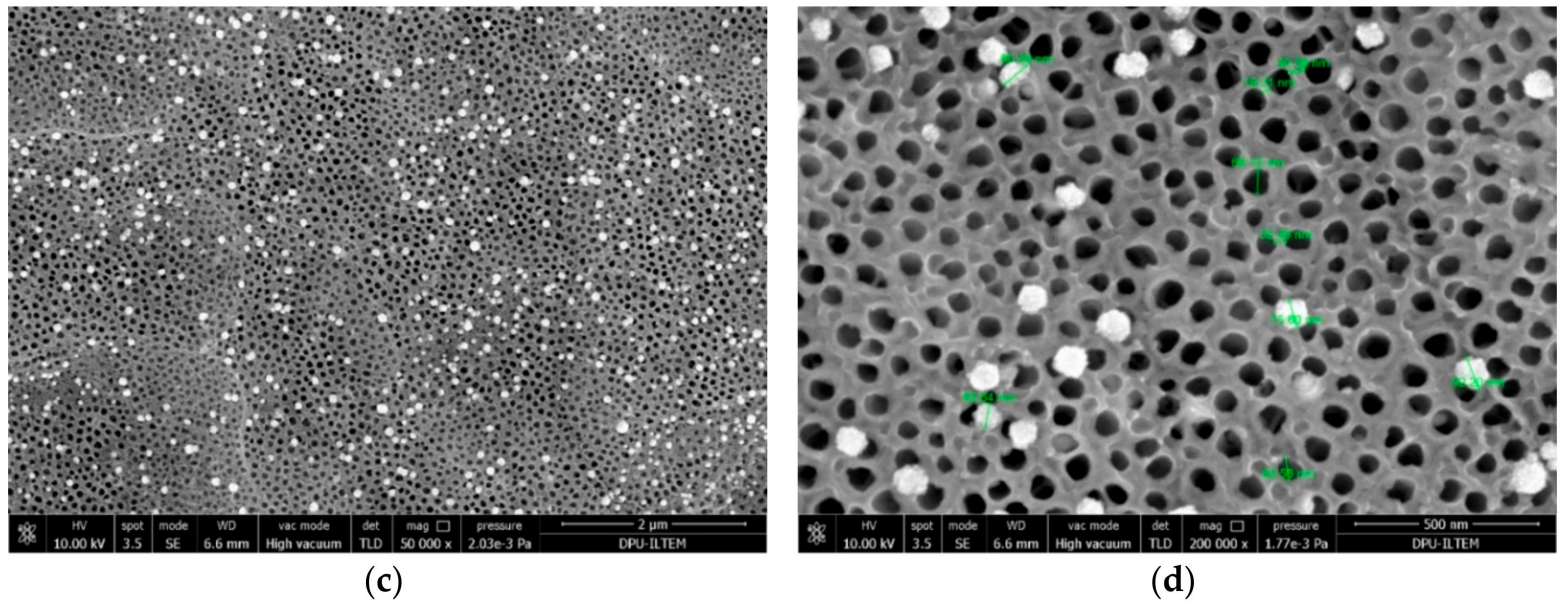
SEM images of photoanodes: Ti/TiO_2_NTEG-3h-500 (**a**,**b**); and Ti/TiO_2_NTEG-3h-500-Pt-4cycles (**c**,**d**). Magnifications: 50,000× (**a**,**c**); and 200,000× (**b**,**d**).

The SEM images of both Ti/TiO_2_NTEG-3h-500 and the corresponding Pt loaded anodes are presented in Figure 9. It is possible to notice the platinum nanoparticle on the external surface of the nanotubes. EDX analysis evidenced that the white nanoparticles belong to Pt. Both TiO_2_ nanotubes and Pt nanoparticles located outside of the pores are quite homogeneously distributed. The nanotube wall thickness and inner hole diameter of these electrodes are 35 and 60 nm, respectively (Table 2). The average diameter of the Pt nanoparticles is about 80 nm.

**Figure 10:**
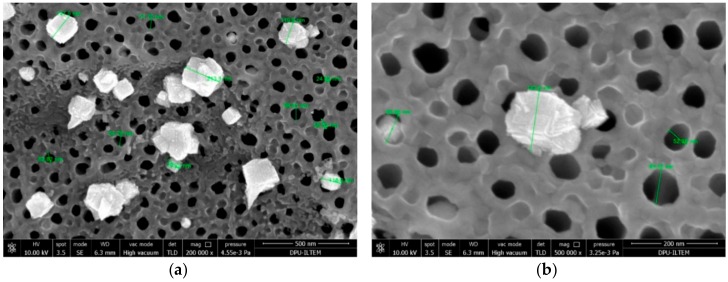
SEM images of Ti/TiO_2_NTEG-3h-500-Pt-25cycles photoanode at magnification: 200,000× (**a**); and 500,000× (**b**).

Figure 10 shows SEM images of Ti/TiO_2_NTEG-3h-500-Pt-25cycles photoanode. Although the TiO_2_ nanotube morphology for this electrode is almost the same of that of Ti/TiO_2_NTEG-3h-500-Pt-4cycles, Pt nanoparticles are quite different in dimensions (ca. twice). In addition, Pt nanoparticle sizes are quite different from each other and they have a heterogeneous distribution in this electrode. This feature indicates that, by increasing the number of cycles, the initially formed small nanoparticles act as crystallization nuclei which grow without the creation of new particles.

The Pt nanoparticle diameter on the surface of Ti/TiO_2_NTEG-3h-500-Pt-25cycles electrode is about 150 nm (Table 2). It is clear from SEM image that Pt nanoparticles are in cubic form. In our previous study, TiO_2_ nanotube surfaces were loaded with Pt by photoreduction method and much smaller and heterogeneously distributed Pt nanoparticles were obtained [18].

**Figure 11:**
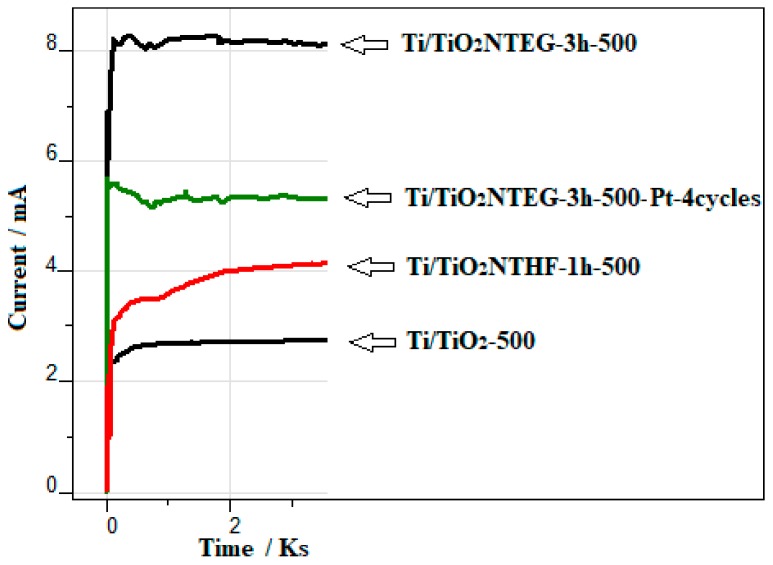
The photocurrent values of electrodes, prepared by different methods, in the same medium in which PEC paraquat degradation reactions were performed.

Figure 11 shows the photocurrent values of electrodes, prepared by different methods, in the same medium in which PEC paraquat degradation reactions were performed. The lowest photocurrent value is attributed to the Ti/TiO_2_-500 photoanode obtained by the thermal oxidation method with the least effective surface area. The photocurrent values of the electrode prepared in HF medium is a little higher than that of Ti/TiO_2_-500 because of its higher effective surface area. Therefore, the electrode with very long nanotube (Ti/TiO_2_NTEG-3h-500) showed the highest photocurrent values, as expected. However, Pt nanoparticles loaded to the Ti/TiO_2_NTEG-3h-500 surface reduced the absorbed amount of light on TiO_2_ surface, resulting in a decrease in the photocurrent value.

**Figure 12:**
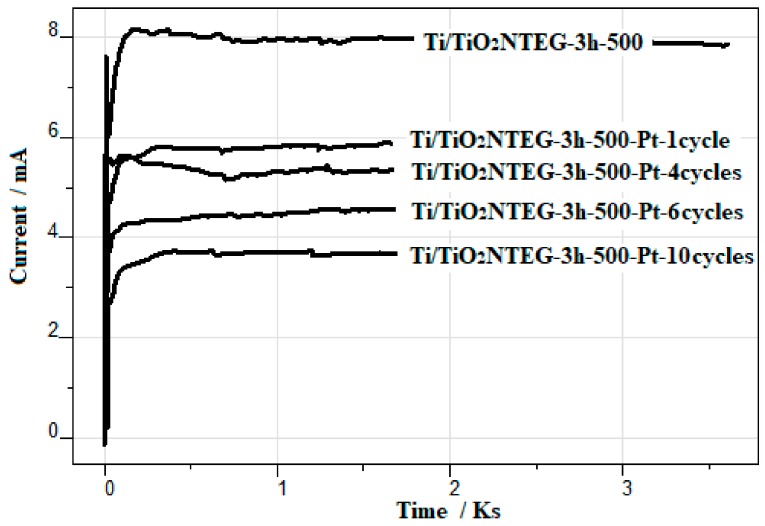
The photocurrent values of Ti/TiO_2_NTEG-3h-500 and Ti/TiO_2_NTEG-3h-500-Pt-Xcycles electrodes in the same medium in which PEC paraquat degradation reactions were performed.

Figure 12 shows the photocurrent values of Ti/TiO_2_NTEG-3h-500-Pt-Xcycles by the choronoamperometric method. As the amount of loaded Pt amount on the nanotube surface increases, the photocurrent values decrease gradually. This could be due to the absorbed photon amount on TiO_2_ surface which decreases by increasing Pt amount. 

### 3.2. Photoelectrocatalytic Activity Results

Preliminary experiments showed that 1.0 V was the optimum bias for the PEC degradation of paraquat (see Figure S13), thus all PEC and EC experiments were performed at 1.0 V.

Table 3 shows the results of PEC degradation of paraquat using Ti/TiO_2_-500 and Ti/TiO_2_NTHF-1h-Y photoanodes. Except for Ti/TiO_2_NTHF-1h-750, all Ti/TiO_2_NTHF-1h-Y photoanodes showed higher PEC activity than Ti/TiO_2_-500, probably because the nanotube structured electrodes have higher surface area. Ti/TiO_2_NTHF-1h-750 did not show any PEC activity, due to the very high calcination temperature, which probably caused the loss of hydroxyl groups responsible of oxygen adsorption [9]. Oxygen is necessary, as it is an electron acceptor for the initial step of the redox reaction [36]. Ti/TiO_2_NT-1h-500 showed the highest paraquat conversion for 3 h PEC reaction time. Indeed, its conversion is almost two-fold higher than Ti/TiO_2_-500 (30% vs. 17%).

Table 4 shows PEC paraquat degradation results of Ti/TiO_2_NTEG-Xh-500, Ti/TiO_2_NTHF-1h-500 and Ti/TiO_2_NTHF-6h-500 electrodes. Results of representative PEC experiments are shown in Figure S14. Ti/TiO_2_NTEG-1h-500 exhibited almost three times higher conversion (ca. 86% versus 30%) than Ti/TiO_2_NTHF-1h-500 for 3h PEC reaction time. This result is due to the formation of longer nanotube structures in the EG medium, instead of in HF, as SEM images suggest.

Nanotube pores are located in vertical position with respect to the irradiation. Therefore, the light could reach the inside of the pores. For this reason, anodes with nanotube structured TiO_2_ showed much more activity compared to non-nanotube structured one (Ti/TiO_2_-500). In addition, the anodes with long nanotubes showed higher PEC activity than shorter ones. However, due to the diffraction and reflection of photons inside of the tube, the intensity of light decreases as it travels through the tube [37].

The effect of the anodic oxidation time on the preparation of Ti/TiO_2_NTEG-Xh-500 electrodes was investigated and the best activity was achieved by Ti/TiO_2_NTEG-3h-500 photoanode. Figure S15 shows UV-Vis absorbance values of the samples taken from the reaction medium at fixed times during PEC degradation of paraquat by using the Ti/TiO_2_NTEG-3h-500 photoanode. As it can be noticed by the observation of the SEM results (see Table 2), the length of the TiO_2_ nanotubes increased considerably by increasing the anodic oxidation time. A drawback can be the fact that, for very long nanotubes, the light cannot irradiate the whole internal surface. Indeed, the results of Ti/TiO_2_NTEG-4h-500 and Ti/TiO_2_NTEG-3h-500 are similar. In addition, the Ti/TiO_2_NTEG-6h-500 electrode showed a lower activity, probably because parts of the nanotubes are covered by a layer of TiO_2_, thus causing a decrease of the active surface area as shown in the SEM image (Figure 8d). Furthermore, since its nanotube length is very high (11 μm), the mass transfer of paraquat at the bottom of the tube could be limited. 

The comparison between electrodes prepared in different solvents (e.g., EG or HF solution) shows that, by using EG, a higher activity was obtained because longer nanotubes formed.

**Figure 13:**
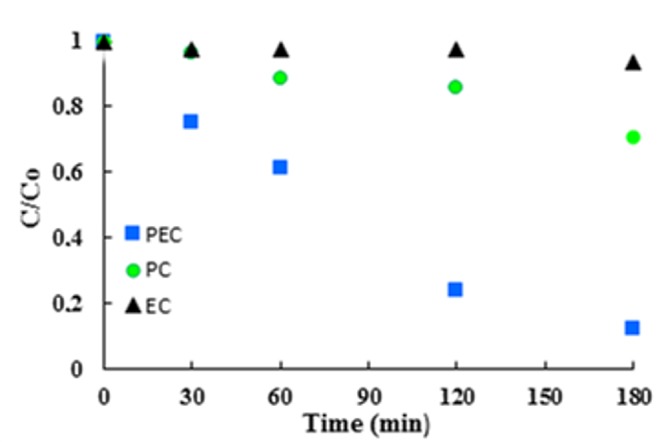
Experimental results of PC, EC and PEC degradation of paraquat by using Ti/TiO_2_NTEG-6h-500 photoanode. [paraquat]: 37.4 μM, applied potential vs. Ag/AgCl: 1.0 V.

Moreover, it can be noticed that runs carried out by using only PC and EC showed very low activities (Table 4 and Figure 13). Therefore, it is evident that photoelectrocatalysis shows a high synergy between PC and EC methods, by reducing the recombination rate of the electron–hole pairs formed by UV irradiation. In other words, by decreasing the recombination rate, the possibility of the interaction of electron–hole pairs with suitable species increases.

Table 5 reports the results of PEC paraquat degradation experiments carried out by using Ti/TiO_2_NTEG-3h-500-Pt-Xcycles photoanodes. By increasing the number of cycles used for Pt loading, the Pt amount increased together with the size of Pt particles, as shown in SEM images. Ti/TiO_2_NTEG-3h-500-4cycles photoanode showed the best performance (75% conversion), probably due to an optimal Pt dispersion. With bigger Pt nanoparticle size, the activity decreased probably due to a reduction of the effective surface area of the photoanode. Non-platinized one (Ti/TiO_2_NTEG-3h-500) showed 60% conversion for 1 h reaction time.

## 4. Conclusions

Effective nanotube structured TiO_2_ on Ti plate photoanodes in ethylene glycol medium (Ti/TiO_2_-NTEG) was prepared, characterized and used for photoelectrocatalytic degradation of paraquat, which is one of the most used herbicides. Thermally oxidized TiO_2_ on Ti plate (Ti/TiO_2_-500) and nanotube structured TiO_2_ on Ti plates prepared in HF medium (Ti/TiO_2_-NTHF) were also prepared and used for comparison. Ti/TiO_2_-NTEG photoanodes were also loaded by Pt nanoparticles by cyclic voltammetry method. The effects of nanotube length and Pt amount of photoanodes on the activity were investigated. The obtained results show that Ti/TiO_2_-NTEG photoanodes consists of very long TiO_2_ nanotubes which raise the activity, therefore they showed much higher PEC activity than other type of used electrodes. 

According to XRD analysis, all Ti/TiO_2_-NTEG-Xh-Y electrodes are in anatase phase with negligible amount of rutile whose amount increased by increasing thermal treatment temperature. SEM and XRD results show that loaded Pt in photoanode is in the metallic form and in cubic structure. In addition, Pt nanoparticles grow with increasing number of Pt loading cycles.

The primary particle sizes of the anatase peak of all Ti/TiO_2_-NTEG electrodes are close to each other and are about 35 nm. Interestingly, this value is approximately the same the wall thickness of the TiO_2_ nanotubes of Ti/TiO_2_-NTEG photoanodes. We also found a linear correlation between nanotube length and XRD peak intensity. 

Photocurrent values of Ti/TiO_2_NTEG photoanode are higher than that of Ti/TiO_2_NTHF. Moreover, by increasing loaded Pt amount on Ti/TiO_2_NTEG photoanode, photocurrent values decrease linearly due to the reduced absorbed photon amount on the TiO_2_ surface.

Ti/TiO_2_-NTEG-6h-500 has the longest nanotube, however a portion of the nanotube surface is covered by TiO_2_ layers. These layers had a detrimental effect on the photoelectrocatalytic activity because they reduced the effective surface area of the material.

Ti/TiO_2_-NTEG-3h-500-Pt-4cycles photoanode showed highest PEC activity for paraquat degradation. We obtained a significant synergy for PEC reaction of paraquat as the PC oxidation reaction was slow and especially almost no EC activity was obtained. Indeed, PEC process reduced the recombination rate of the electron–hole pairs formed by UV irradiation on the photoanode surface.

## Figures and Tables

**Table 1:** Crystal phase, peak areas (101) of anatase and rutile phases, and primary particle sizes of the prepared anodes.

Electrode	Crystal Phase	Peak Area of Anatase (101)	Primary Particle Size (nm) of Anatase	Primary Particle Size (nm) of Rutile	Primary Particle Size (nm) of Pt
Ti/TiO_2_NTEG-3h-450	A	423	37		
Ti/TiO_2_NTEG-3h-500-Pt-25cycles	A + R	450	33		28
Ti/TiO_2_NTEG-3h-550	A + R	477	35	43	
Ti/TiO_2_NTEG-1h-500	A + R	194	35	22	
Ti/TiO_2_NTEG-6h-500	A + R	558	38	37	
Ti/TiO_2_NTHF-6h-500	A + R	48	32	23	

**Table 2:** Average wall thickness and internal diameters of nanotubes on the anodes evaluated by SEM imagines.

Electrode	Wall Thickness (nm)	Internal Diameter (nm)	Tube Length (μm)	Pt Nanoparticle Diameter (nm)
Ti/TiO_2_NTEG-1h-500	35	43	1.7	
Ti/TiO_2_NTEG-2h-500	30	47		
Ti/TiO_2_NTEG-3h-500	35	60		
Ti/TiO_2_NTEG-4h-500	35	47	9.8	
Ti/TiO_2_NTEG-6h-500	20	80	11	
Ti/TiO_2_NTHF-6h-500	14	90		
Ti/TiO_2_NTHF-6h-650	30	75	1.0	
Ti/TiO_2_NTEG-3h-500-Pt-4cycles	35	60		80
Ti/TiO_2_NTEG-3h-500-Pt-25cycles	40	60		150

**Table 3:** PEC degradation results of paraquat by using Ti/TiO_2_-500 and Ti/TiO_2_NTHF-1h-Y electrodes. [paraquat]: 37.4 μM, Applied potential vs. Ag/AgCl: 1.0 V.

Photoanode	Calcination Temperature (°C)	Conversion for 3 h (%)
Ti/TiO_2_-500	500	17
Ti/TiO_2_NTHF-1h-400	400	28
Ti/TiO_2_NTHF-1h-500	500	30
Ti/TiO_2_NTHF-1h-600	600	23
Ti/TiO_2_NTHF-1h-650	650	26
Ti/TiO_2_NTHF-1h-750	750	0

**Table 4:** The results of PEC, PC and EC paraquat degradation by using Ti/TiO_2_NTEG-Xh-500 photoelectrodes. [paraquat]: 37.4 μM, applied potential vs. Ag/AgCl: 1.0 V.

Photoanode	Method	Anodic Oxidation Time	Conversion for 1 h (%)	Conversion for 3 h (%)
Ti/TiO_2_NTEG-1h-500	PEC	1	43	86
Ti/TiO_2_NTEG-2h-500	PEC	2	52	93
Ti/TiO_2_NTEG-3h-500	PEC	3	60	98
Ti/TiO_2_NTEG-4h-500	PEC	4	62	95
Ti/TiO_2_NTEG-6h-500	PEC	6	50	90
Ti/TiO_2_NTEG-6h-500	PC	6	7	26
Ti/TiO_2_NTEG-6h-500	EC	6	0	1
Ti/TiO_2_NTHF-1h-500	PEC	1	8	30
Ti/TiO_2_NTHF-6h-500	PEC	6	9	48

**Table 5:** PEC experiment results of Ti/TiO_2_NTEG-3h-500-Pt-Xcycles photoanodes. [paraquat]: 37.4 μM, applied potential vs. Ag/AgCl: 1.0 V.

Photoanode	Cycle Count	Conversion for 1 h (%)
Ti/TiO_2_NTEG-3h-500	-	60
Ti/TiO_2_NTEG-3h-500-Pt-1cycle	1	49
Ti/TiO_2_NTEG-3h-500-Pt-3cycles	3	60
Ti/TiO_2_NTEG-3h-500-Pt-4cycles	4	75
Ti/TiO_2_NTEG-3h-500-Pt-5cycles	5	68
Ti/TiO_2_NTEG-3h-500-Pt-6cycles	6	60
Ti/TiO_2_NTEG-3h-500-Pt-7cycles	7	52
Ti/TiO_2_NTEG-3h-500-Pt-10cycles	10	46
Ti/TiO_2_NTHF-3h-500-Pt-25cycles	25	42

## Supplementary Materials

The following are available online at http://www.mdpi.com/1996-1944/11/9/1715/s1, Figure S1: The photo of Ti/TiO_2_-500 photoanode, Figure S2: Experimental setup used for anodic oxidation, Figure S3: The photos of Ti/TiO_2_NTHF-X-Y photoanodes, Figure S4: The photos of Ti/TiO_2_NTEG-X-Y photoanodes, Figure S5: Voltammograms obtained during Pt nanoparticle loading on Ti/TiO_2_NTEG-3h-500 electrode by CV until 4 cycles. The first cycle is black, the second is green, the third is red and the fourth is blue, Figure S6: SEM images of Ti/TiO_2_NTHF-6h-650 photoanode (magnification: 1000× (a), and 5000× (b)), Figure S7: PEC experiment system (up) and the spectra of the used UV fluorescent lamp (below), Figure S8: XRD patterns of Ti/TiO_2_NTEG500-3h-Pt-25cycles electrode, Figure S9: SEM image of Ti/TiO_2_-500 photoanode. Magnification: 50,000× Figure S10: SEM image of Ti/TiO_2_NTHF-1h-500 photoanode (magnification: 250,000×), Figure S11: SEM images of Ti/TiO_2_NTEG-4h-500 photoanode. Cross section view (magnification: 10,000×), Figure S12: SEM images of Ti/TiO_2_NTEG-6h-500 photoanode. Cross section view (magnification: 150,000×). Bottom view (magnification: 20,000×), Figure S13: The conversion values for PEC paraquat degradation for 1 (blue) and 3 (green) hours of reaction time at different voltage values in the presence of Ti/TiO_2_NTEG-3h-500 electrode, Figure S14. The PEC experiment results of Ti/TiO_2_-500 (), Ti/TiO_2_NTHF-1h-500 (), Ti/TiO_2_NTEG-1h-500 (), Ti/TiO_2_NTEG-3h-500 () and Ti/TiO_2_NTEG-6h-500 () for paraquat degradation. Potential: 1V, Figure S15. UV-Vis absorbance values of the samples taken from the reaction medium at fixed times during PEC degradation of paraquat (37.4 μM) at 1 V by using the Ti/TiO_2_NTEG-3h-500 photoanode.
