# Supplementary material for: Photoelectrocatalytic Degradation of Paraquat by Pt Loaded TiO2 Nanotubes on Ti Anodes
Source: Materials (Basel). 2018 Sep 13;11(9):1715. doi: 10.3390/ma11091715 (PMC6164922; doi:10.3390/ma11091715)
Supplement: Supplementary file 1 [file materials-11-01715-s001.pdf]

Supplementary Materials: Photoelectrocatalytic Degradation of Paraquat by Pt Loaded TiO2 Nanotubes on Ti Anodes

Levent Özcan [1,*], Turan Mutlu [2] and Sedat Yurdakal [2,*]

1 Biyomedikal Mühendisliği Bölümü, Mühendislik Fakültesi, Afyon Kocatepe Üniversitesi, Ahmet Necdet Sezer Kampüsü, 03200 Afyonkarahisar, Turkey

2 Kimya Bölümü, Fen-Edebiyat Fakültesi, Afyon Kocatepe Üniversitesi, Ahmet Necdet Sezer Kampüsü, 03200 Afyonkarahisar, Turkey; turan.mutlu@redokslab.com

* Correspondence: leventozcan@aku.edu.tr; sedatyurdakal@gmail.com

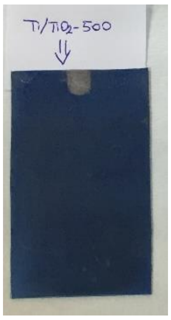

Figure S1. The photo of Ti/TiO_2-500 photoanode.

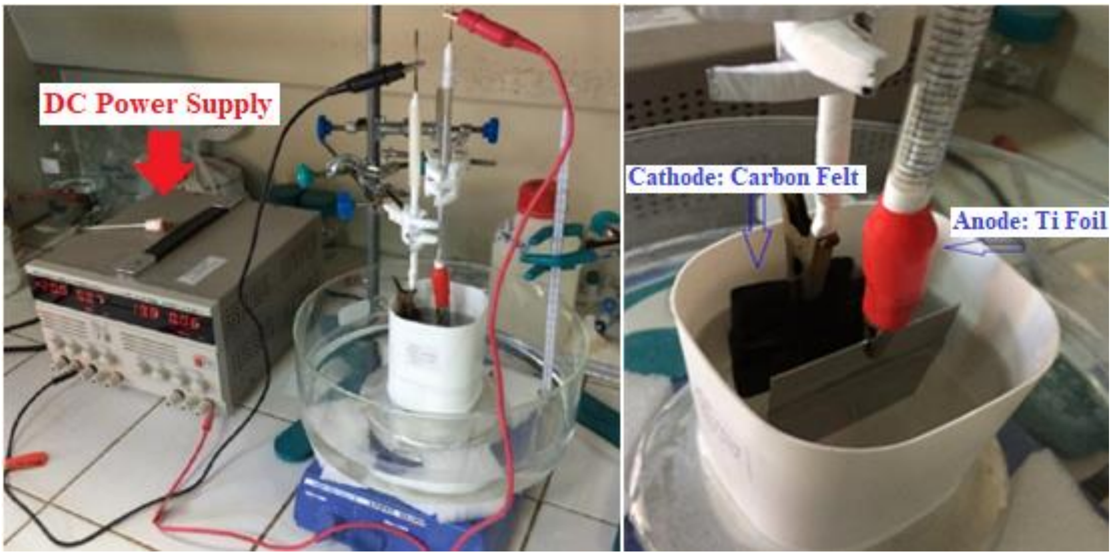

Figure S2. Experimental setup used for anodic oxidation.

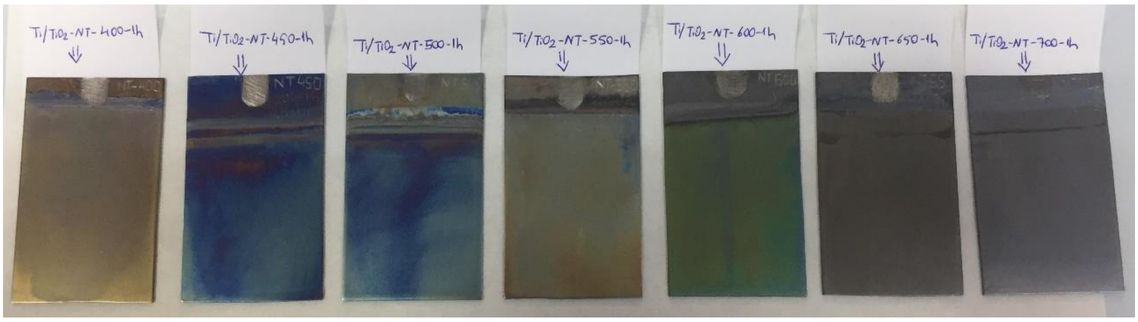

Figure S3. The photos of Ti/TiO_2NTHF-X-Y photoanodes.

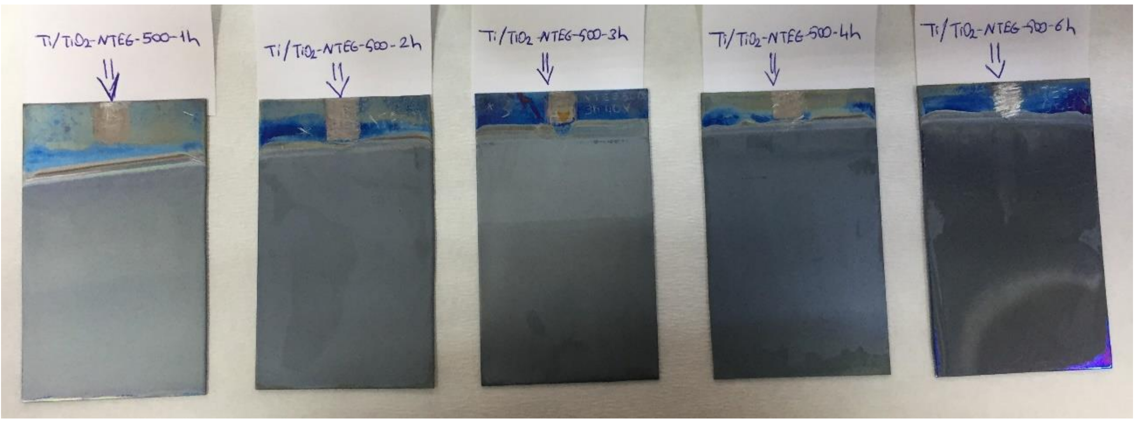

Figure S4. The photos of Ti/TiO_2NTEG-X-Y photoanodes.

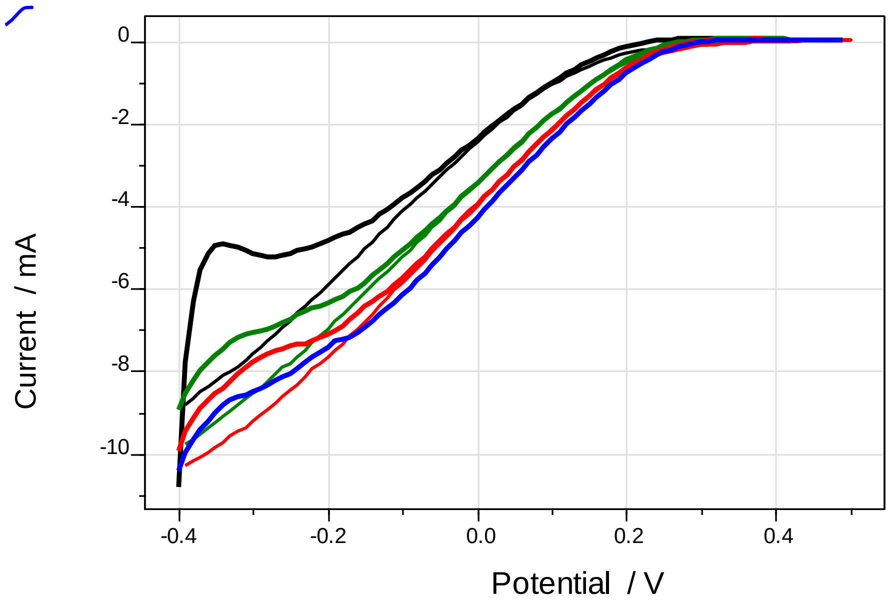

Figure S5. Voltammograms obtained during Pt nanoparticle loading on Ti/TiO_2NTEG-3h-500 electrode by CV until 4 cycles. The first cycle is black, the second is green, the third is red and the fourth is blue.

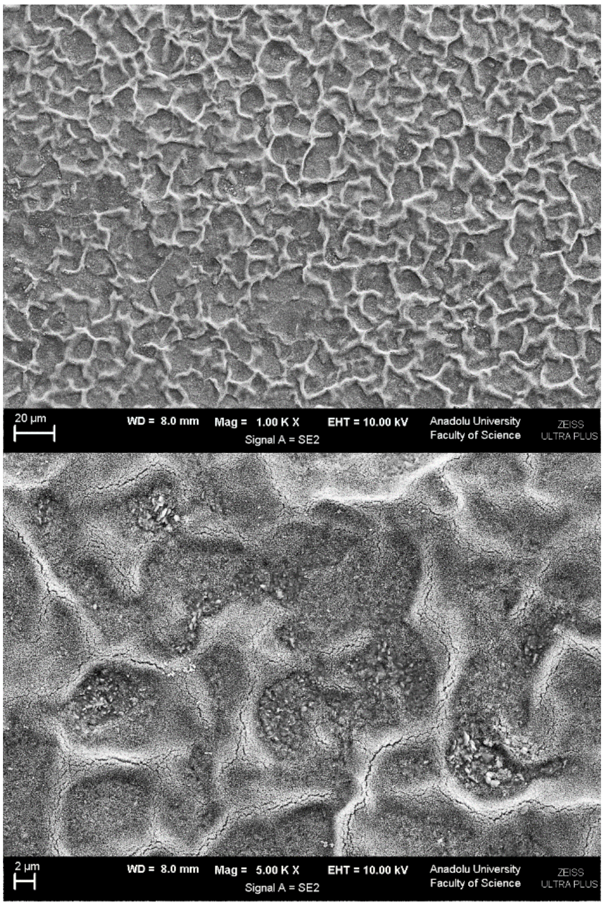

Figure S6. SEM images of Ti/TiO_2NTHF-6h-650 photoanode (magnification: 1000× (**a**), and 5000× (**b**)).

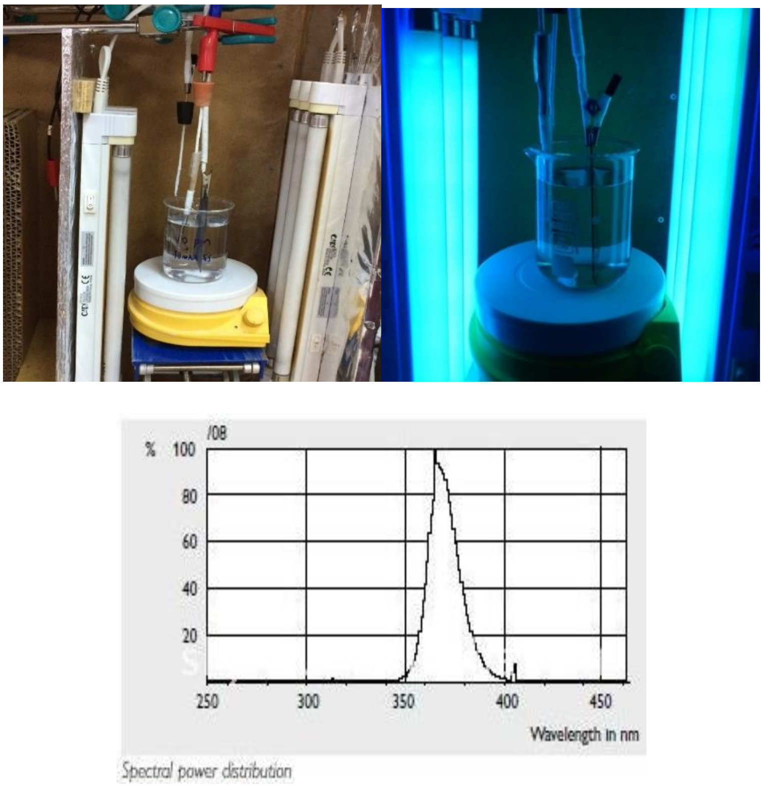

Figure S7. PEC experiment system (up) and the spectra of the used UV fluorescent lamp (below).

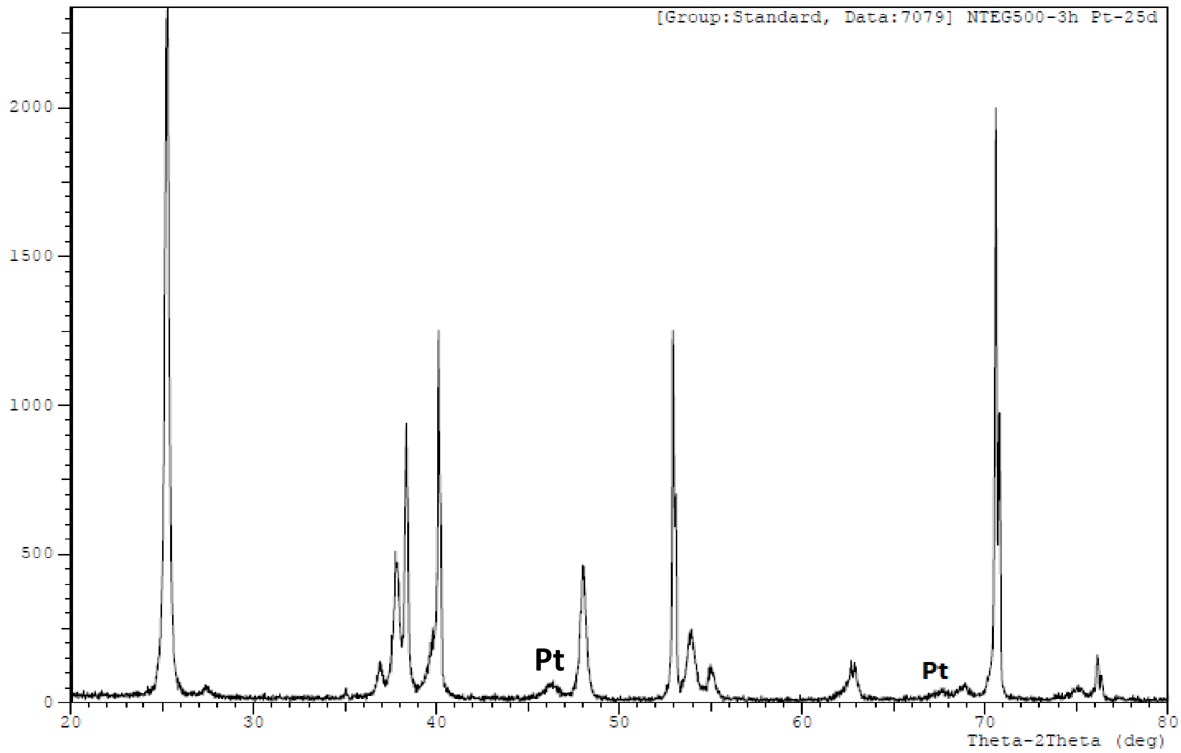

Figure S8. XRD patterns of Ti/TiO_2NTEG500-3h-Pt-25cycles electrode.

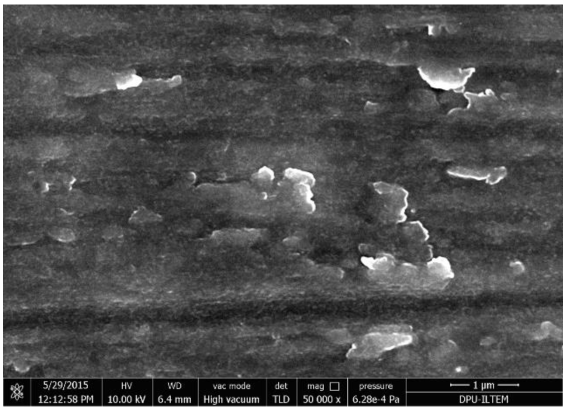

Figure S9. SEM image of Ti/TiO_2-500 photoanode. Magnification: 50000×. Reprinted with permission from [1]. Copyright 2017 Elsevier.

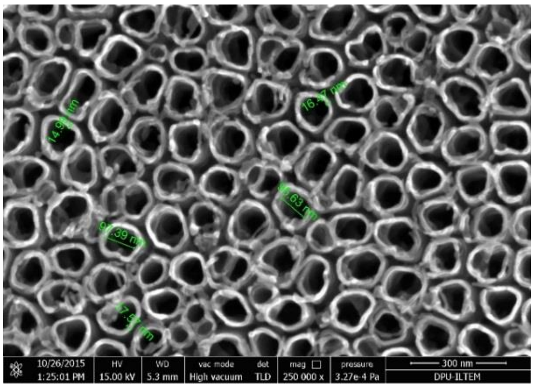

Figure S10. SEM image of Ti/TiO_2NTHF-1h-500 photoanode (magnification: 250000×). Reprinted with permission from [1]. Copyright 2017 Elsevier.

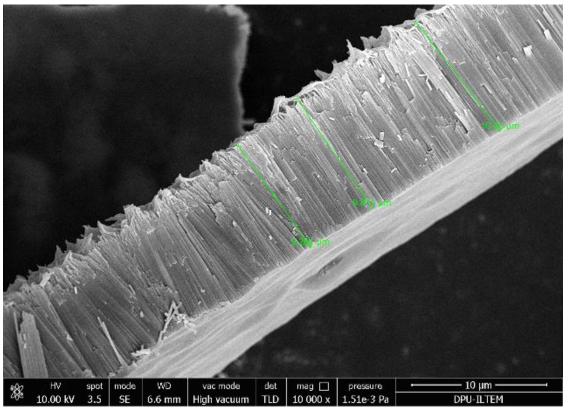

Figure S11. SEM images of Ti/TiO2NTEG-4h-500 photoanode. Cross section view (magnification: 10000×).

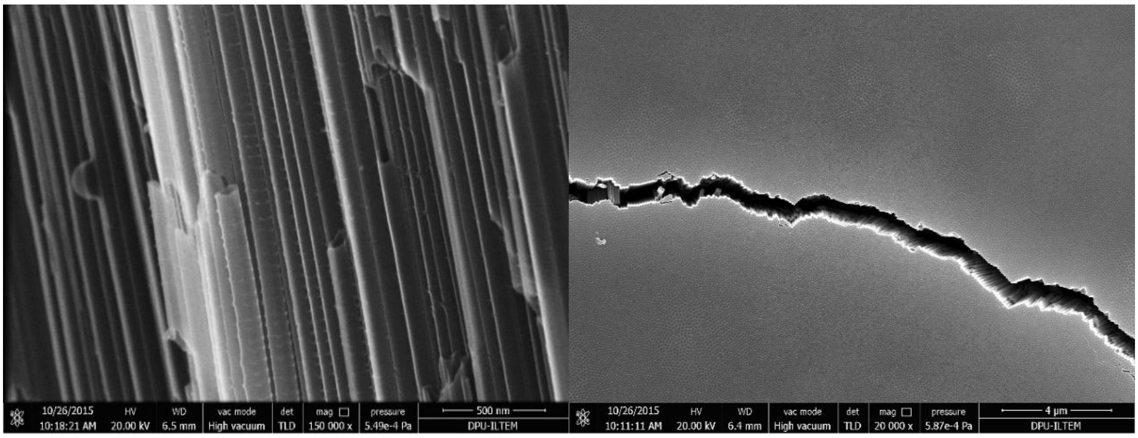

Figure S12. SEM images of Ti/TiO2NTEG-6h-500 photoanode. Cross section view (magnification: 150000X). Bottom view (magnification: 20000×).

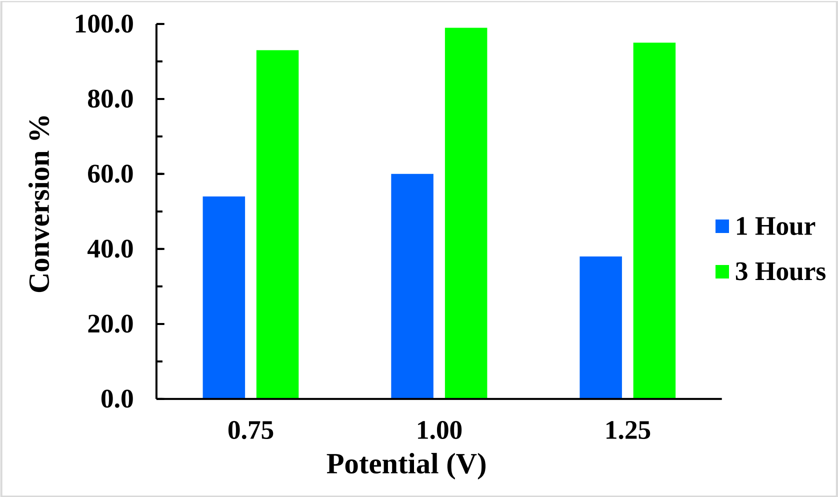

Figure S13. The conversion values for PEC paraquat degradation for 1 (blue) and 3 (green) hours of reaction time at different voltage values in the presence of Ti/TiO2NTEG-3h-500 electrode.

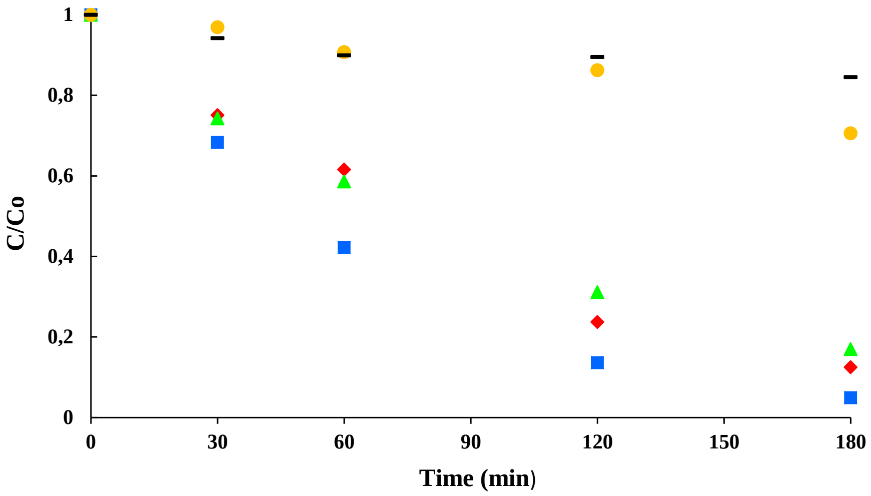

Figure S14. The PEC experiment results of Ti/TiO_2-500 (▬), Ti/TiO_2NTHF-1h-500 (●), Ti/TiO_2NTEG-1h-500 (▲), Ti/TiO_2NTEG-3h-500 (■) and Ti/TiO_2NTEG-6h-500 (◆) for paraquat degradation. Applied potential: 1V.

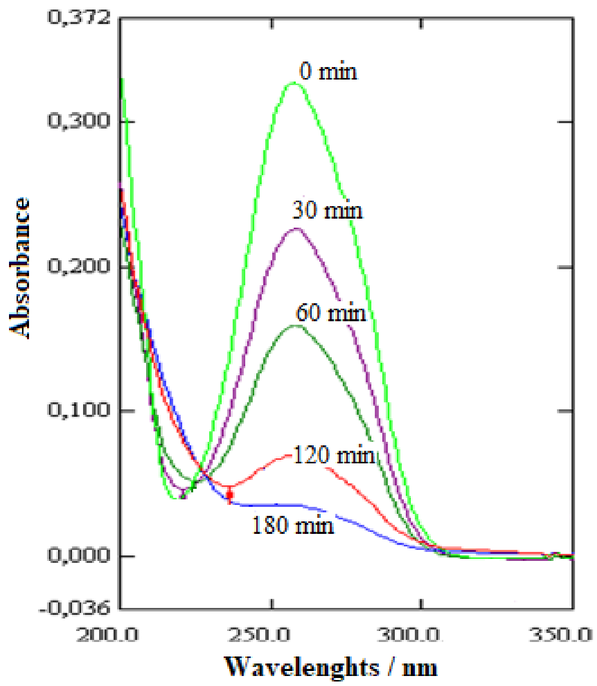

Figure S15. UV-Vis absorbance values of the samples taken from the reaction medium at fixed times during PEC degradation of paraquat (37.4 μM) at 1 V by using the Ti/TiO_2NTEG-3h-500 photoanode.

References

1. Özcan, L.; Yalçın, P.; Alagöz, O.; Yurdakal, S. Selective photoelectrocatalytic oxidation of 5-(hydroxymethyl)-2-furaldehyde in water by using Pt loaded nanotube structure of TiO2 on Ti photoanodes. *Catal. Today* **2017**, *281*, 205–213.

© 2018 by the authors. Submitted for possible open access publication under the terms and conditions of the Creative Commons Attribution (CC BY) license (http://creativecommons.org/licenses/by/4.0/).
